# Strains on Resident Doctors’ Workloads: An Audit of On-Call Room Conditions and Driving Distances

**DOI:** 10.1192/bjo.2026.11660

**Published:** 2026-06-30

**Authors:** Youssef Ashour, Mohamed Elsout, Omar Elsaka

**Affiliations:** 1 Aneurin Bevan University Health Board, Cardiff, United Kingdom; 2 Cwm Taf Morgannwg University Health Board, Cardiff, United Kingdom; 3 Ty Glyn Ebwy and Ty gwyn Hall, Elysium Healthcare limited, Cardiff, United Kingdom

## Abstract

**Aims:**

The aim of this auditis to assess the adequacy of on-call room facilities and their impact on resident doctors’ rest and recovery during shifts. It also sought to evaluate the challenges and safety risks associated with driving long distances during and after on-call duties.

**Methods:**

Data were collected through direct observation and surveys completed by participating resident doctors. The audit assessed cleanliness, availability of rest facilities, access to kitchen and bathroom amenities, and perceived impact on fatigue and driving safety. Driving distances between the hospital and doctors’ homes were recorded and analysed. Surveys also explored how often doctors felt tired when driving after on-call shifts and their perceived ability to drive home safely. Data were collected from seven resident doctors covering shifts between 17 August 2024 and 20 September 2024.

**Results:**

The audit identified significant deficiencies in on-call room conditions. Rooms were frequently unclean, infested with insects, and lacked basic hygiene measures such as bed linen. Many rooms were used as day offices, limiting opportunities for rest. Additional issues included lack of water access in kitchen areas and poor lighting. These conditions were associated with increased fatigue and stress among resident doctors.

Driving analysis showed a total of 626.3 miles driven during recorded journeys, with 247.4 miles driven when returning home after on-call shifts. Survey responses indicated high levels of fatigue related to driving: doctors reported feeling occasionally tired on 6 shifts, frequently tired on 17 shifts, and very frequently tired on 15 shifts. Regarding the ability to drive home, responses were “Yes” for 18 shifts, “No” for 4 shifts, and “Maybe” for 16 shifts.

**Conclusion:**

The findings underscore the need to enhance on-call room facilities to ensure adequate rest and mitigate fatigue-related risks. Improvements should align with BMA guidance, including provision of dedicated, hygienic rest areas that are not used for clinical work. Addressing long driving distances and post-shift commuting risks is also crucial to enhancing resident doctors’ safety and well-being.

